# Relationship between oxide identity and electrocatalytic activity of platinum for ethanol electrooxidation in perchlorate acidic solution

**DOI:** 10.1038/s42004-023-00908-3

**Published:** 2023-05-29

**Authors:** Xinyu You, Jiaxing Han, Vinicius Del Colle, Yuqiang Xu, Yannan Chang, Xiao Sun, Guichang Wang, Chen Ji, Changwei Pan, Jiujun Zhang, Qingyu Gao

**Affiliations:** 1 College of Chemical Engineering, China University of Mining and Technology, 221116 Xuzhou, People’s Republic of China; 2 Department of Chemistry, Federal University of Alagoas-Campus Arapiraca, Av. Manoel Severino Barbosa s/n, Arapiraca, AL 57309-005 Brazil; 3 Department of Chemistry, Nankai University, 300071 Tianjin, People’s Republic of China; 4 School of Materials Science and Engineering, Fuzhou University, 350108 Fuzhou, People’s Republic of China

**Keywords:** Electrocatalysis, Reaction kinetics and dynamics, Electrocatalysis, Heterogeneous catalysis

## Abstract

Water and its dissociated species at the solid‒liquid interface play critical roles in catalytic science; e.g., functions of oxygen species from water dissociation are gradually being recognized. Herein, the relationship between oxide identity (PtOH_ads_, PtO_ads_, and PtO_2_) and electrocatalytic activity of platinum for ethanol electrooxidation was obtained in perchlorate acidic solution over a wide potential range with an upper potential of 1.5 V (reversible hydrogen electrode, RHE). PtOH_ads_ and α-PtO_2_, rather than PtO_ads_, act as catalytic centers promoting ethanol electrooxidation. This relationship was corroborated on Pt(111), Pt(110), and Pt(100) electrodes, respectively. A reaction mechanism of ethanol electrooxidation was developed with DFT calculations, in which platinum oxides-mediated dehydrogenation and hydrated reaction intermediate, geminal diol, can perfectly explain experimental results, including pH dependence of product selectivity and more active α-PtO_2_ than PtOH_ads_. This work can be generalized to the oxidation of other substances on other metal/alloy electrodes in energy conversion and electrochemical syntheses.

## Abstract (web summary)

Interfacial water and its dissociated species play key roles in aqueous electrocatalysis. Here, the authors study the ethanol electrooxidation on platinum electrodes at potentials up to 1.5 V vs. RHE and find that the oxygen species stemming from water, PtOH_ads_, and α-PtO_2_ act as catalytic centers.

## Introduction

In-depth exploration of the dynamic mechanism at the water–solid interface advances the development and utility of heterogenous catalysis in electrochemical^1–4^, chemical^5–9^, and photochemical reactions^10^. The key role of interfacial water for charge transfer at the water–solid interface is recognized in disparate systems^11^. For heterogeneous oxidation reactions, the promotion^5–8,12^ and inhibition^9^ effects of interfacial water on the catalyst surface were reported. In electrochemical reactions, the structures, orientations, and dissociation of interfacial waters depend on the applied potential, electrode composition, and solutes in the aqueous solution^13–15^. The H and O atoms of interfacial water point toward the surface at different potentials^14^, which aids H- and O-related reactions. Intuitively, H_2_O acts as a source of H/O adsorbates and promotes oxidation^16^ and reduction^17^ of substrates. However, adsorbed oxygenated species inhibit reactions in many cases^18^.

Platinum is known as the most active element for electrochemical reactions and remains the benchmark for electrocatalyst materials^3^. Potential-dependent Pt oxides and their structures were identified on single-crystal and polycrystalline electrodes by many investigators^19–26^, which is believed as one of the great achievements in basic electrochemistry in the past 30 years. Different analytical methods, such as voltammetric methods^19–21^, X-ray photoelectron spectroscopy (XPS)^21^, X-ray absorption spectroscopy (XAS)^22^, X-ray reflection/diffraction /scattering^22–25^, and Raman spectroscopy^26^, have been used to identify surface Pt oxides (PtOH_ads_, PtO_ads_, and PtO_2_) and their dependence on potential. Electrosorption of OH_ads_ on Pt at potentials lower than 0.95 V (vs. standard hydrogen electrode, SHE) constitutes the initial stage of Pt electrooxidation^21,22^, in which the “butterfly” region of CVs for Pt(111) in HClO_4_ solution contains random adsorption in the broad region followed by the formation of a transient *p* (2 × 2) adsorbate phase and a subsequent phase transition to a *p* (1 × 1) adlayer at the sharp peak^24^. The second stage proceeds with the appearance of PtO_ads_ at approximately 0.95 V, followed by the place-exchange (PE) process^23,25^, in which a Pt surface atom leaves its lattice site and oxygen penetrates the metal lattice; this occurs at approximately 1.05 V. After formation of place-exchanged atoms up to the physical limit at approximately 1.15 V^20^, other surface atoms are oxidized and leave Pt surface lattice sites, this leads to 2D Pt oxides^19^^,^ which ultimately grow to form three-dimensional Pt oxides in higher potential^22,26^.

The function of PtOH_ads_ in electrochemical oxidations of small organic molecules (such as CO^27^, methanol^28,29^, and ethanol^30–33^) and other compounds (such as thiosulfate and thiourea^34^) has been studied in previous reports. However, the function and mechanism of Pt oxides at higher potentials, e.g., PtO_ads_ (0.95–1.15 V) and PtO_2_ (>1.15 V) in electrocatalytic oxidations, have rarely been studied. In this work, we systematically explored the corresponding relationship between Pt oxides and the electrooxidation activity of ethanol using online electrochemical-HPLC and verified the promoting effect of Pt oxides with DFT calculations, which gave an atom-scale mechanistic description of oxide function on ethanol oxidation. At the same time, two points are considered here. First, the relationship between oxide identity and electrooxidation activity of ethanol will not be the focus below 0.5 V (RHE); even the PtOH_ads_ can be found on Pt(100) and Pt(111) and polycrystalline Pt in the low potentials^35–37^, the reason of which is that PtOH_ads_ below 0.5 V (RHE) can be displaced by CO produced by partial C-C bond cleavage of ethanol on P(110), Pt(100), and polycrystalline Pt during repeatedly round-trip scan of applied potential^30,32^, resulting in CO adsorption-induced surface deactivation and this adsorbed CO can be oxidized to CO_2_ only above 0.6 V^29^. Second, we will mainly carry out the experimental study and mechanistic analysis involving the oxidation of ethanol and acetaldehyde since monobasic Pt catalyst shows quietly low CO_2_ selectivity^38^.

## Results and discussion

### Potential-dependent oxide identity on Pt nanoparticles in HClO_4_ solutions

**Fig. 1:**
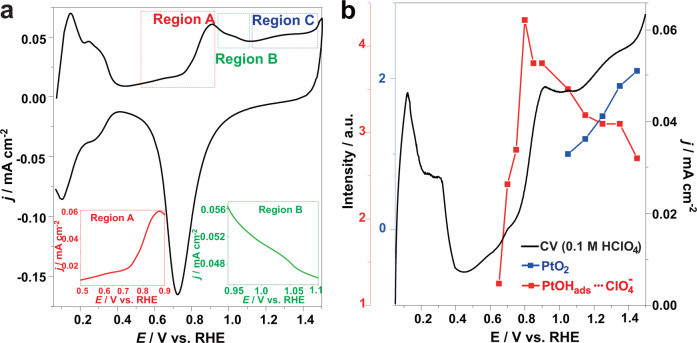
Cyclic voltammogram and potential-dependent in situ SERS for Pt nanoparticles in acidic solution. **a** Cyclic voltammogram of Pt nanoparticles in 0.5 M HClO_4_ solution at potentials between 0.05 V and 1.5 V with a scan rate of 50 mV s^−1^, in which Region A, B, and C represent the potential regions forming PtOH_ads_, PtO_ads_, and α-PtO_2_, respectively. **b** The variations of Raman intensity for the PtOH_ads_ and α-PtO_2_ along the positive voltammetry curve in 0.1 M HClO_4_ electrolyte with a scan rate of 50 mV s^−^^1^. The black, red, and blue solid lines represent the voltammetry curve of Pt, Raman-intensity curves for the PtOH_ads_ and α-PtO_2_, corresponding to the black, red, and blue *Y*-axes, respectively.

Figure 1a shows the cyclic voltammogram (CV) for oxidation of Pt nanoparticles in 0.5 M HClO_4_ solution between 0.05 V and 1.5 V, in which the potential-dependent oxidation processes of Pt are identified based on previous works^22,23,39–42^. Desorption and adsorption of hydrogen at potentials within the range 0.05–0.40 V were followed by the electrical double layer region between 0.40 and 0.60 V. Potentials above 0.60 V constitute the Pt oxide region, where the initial stage of surface oxidation leads to the formation and adsorption of OH_ads_^39,40^. The OH_ads_ coverage in region A increases with potential and begins to convert into O_ads_ at approximately 0.95 V^40^, which corresponds to the start of region B indicated by the place-exchange (PE) process, i.e., a surface Pt atom leaves its lattice site and exchanges with an oxygen species^23,41^. Between 0.95 and 1.15 V, PtOH_ads_ gradually transforms into PtO_ads_ species^22^. At higher potentials, i.e., region C shown in Fig. 1a, higher-order oxides (such as α-PtO_2_) are formed^41,42^.

Figure 1b shows the correspondence between voltammetry data and the Raman spectra. The bands arising at the indicated potentials from 0.65 to 1.45 V are shown in Supplementary Fig. 1 of the Supplementary Information (SI). The band at about 935 cm^−1^ corresponds to the symmetric stretching mode of ClO_4_^−^^43^, which can be regarded as an indirect indicator for the formation of OH_ads_ adlayer^26^ because ClO_4_^−^ is located in the compact part of the interfacial double layer according to the Stark effect^44^. At a potential of 1.15 V, a band begins to appear at ca. 590 cm^−1^; this has been assigned to amorphous α-PtO_2_^26^. The Raman peak intensity for PtOH_ads_···ClO_4_^−^ shows a maximum near the maximum current generated at ca. 0.8 V and decreases with increasing potential, which indicates the formation and coverage of OH_ads_ on the surface of Pt. Additionally, the Raman intensity of the α-PtO_2_ signal emerges at a potential of 1.15 V and increases with potential.

The intensities of the Raman bands qualitatively followed the current for Pt oxidation at potentials higher than 0.65 V, which represented the formation and growth of Pt oxides in this region. The PtOH_ads_ species began to form above 0.60 V, the coverage of which increased with current density. The Raman intensity for PtOH_ads_ decreased with decreasing current density due to the transformation of PtOH_ads_ to PtO_ads_. At a higher potential of approximately 1.15 V, the intensity of the PtOH_ads_ band was severely reduced as current density increased, and the appearance and increased intensity of α-PtO_2_ were caused by the consumption of PtOH_ads_, as shown in Fig. 1b.

### Electrocatalytic activity of Pt oxides for ethanol oxidation on Pt nanoparticles

**Fig. 2:**
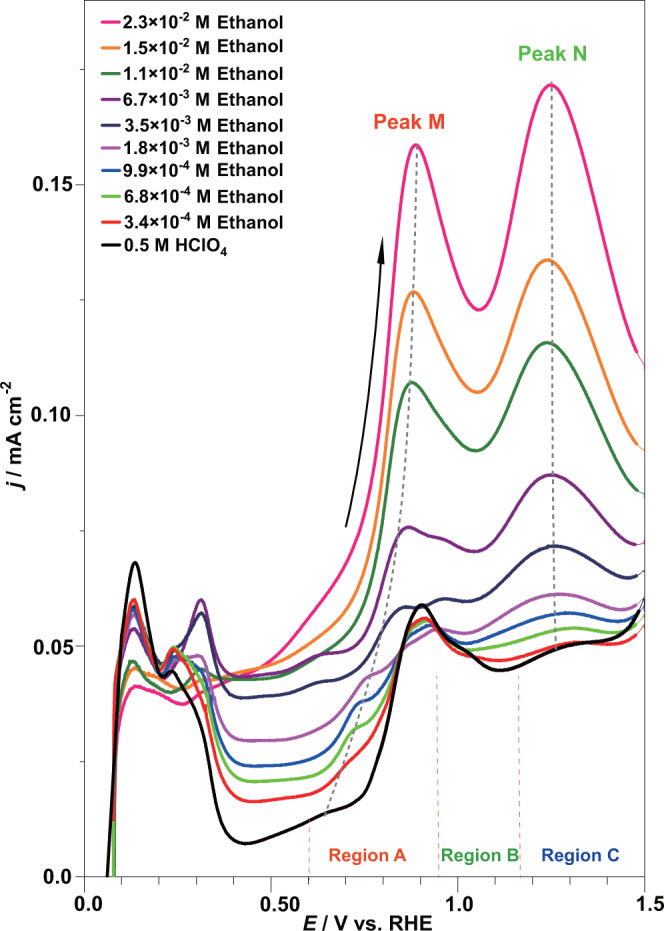
Cyclic voltammetry profiles for ethanol electrooxidation on Pt nanoparticles in 0.50 M HClO_4_ solution. The scan rate was 50 mV s^−^^1^, the black solid line represents the blank solution, and the colorful solid lines represent different concentrations of ethanol.

Figure 2 shows the CV curves for ethanol oxidation on Pt nanoparticles in 0.5 M perchloric acid with different concentrations of ethanol. The current peak M (~0.7 V), beginning from region A related to PtOH_ads_ in the blank solution (black line), shifted to more positive potentials (~0.85 V) with the addition of ethanol, which might be ascribed to competitive adsorption on OH_ads_ with ethanol^45^ and intermediates such as CO^32^. Peak N (~1.3 V), which originated from α-PtO_2_ in the blank solution (black line), was fixed at the same potential upon the addition of ethanol. The current densities of both peak M and peak N increased upon the addition of ethanol, indicating that OH_ads_ and α-PtO_2_ were catalytic active centers. However, the increase in the current density at a potential of 1.05 V located in the valley between peak M and peak N was far smaller than those of peak M and peak N; i.e., PtO_ads_ did not generate the corresponding peak of current density, indicating that PtO_ads_ is an inactive center.

### Further corroboration of oxide activity on three basal crystal planes of Pt in ethanol oxidation

The experimental results described above demonstrate that peak M and peak N for ethanol electrooxidation coincided with the formation and growth of Pt oxides in perchloric acid, indicating that PtOH_ads_ and α-PtO_2_ promote ethanol oxidation at peak M and peak N, respectively; moreover, PtO_ads_ showed almost no corresponding current peak of electrocatalysis in the ethanol oxidation. The atomic arrangement of the polycrystalline electrode is composed of basic single-crystal domains, and the surface of the Pt nanoparticle electrode was characterized by the adsorption of bismuth and germanium, as shown in Supplementary Fig. 2. Calculation results are summarized in Table 1, the Pt nanoparticles consist of 11.69% Pt(100), 16.52% Pt(111), and 71.79% Pt(110) domains. Therefore, the sum of the experimental CV curves with the ratio for three basal crystal planes agreed well with the results for Pt polycrystalline nanoparticles, as shown in Supplementary Fig. 3.

**Table 1:** Fractions of Pt(100), Pt(111), and Pt (110) domains on the Pt nanoparticles.

Crystal plane	Pt(100)	Pt(111)	Pt(110)
Ratio (%)	11.69 ± 0.63	16.52 ± 0.74	71.79 ± 1.37

**Fig. 3:**
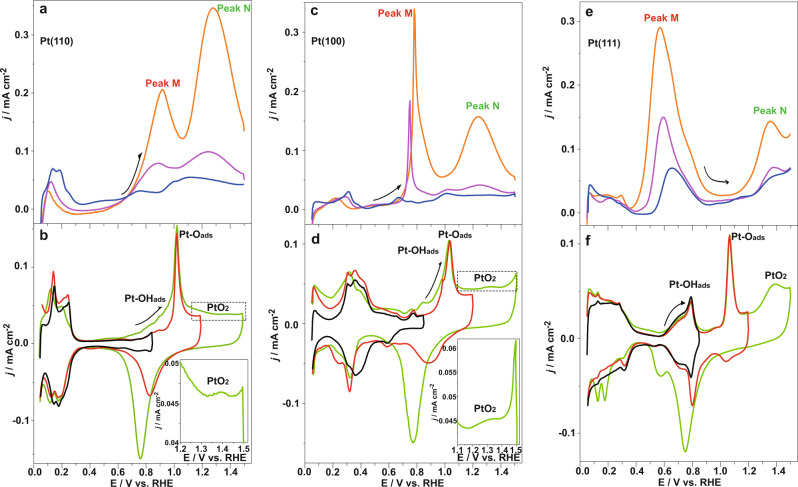
Voltammetric profiles for Pt(110)/(100)/(111) electrodes in ethanol and blank solutions. The j/E profiles of 10^−^^4^ M (blue), 10^−^^3^ M (purple), and 10^−2^ M (orange) for ethanol oxidation (**a**, **c**, **e**) and the CVs of different potential ranges in the blank solution (**b**, **d**, **f**) on the Pt(110), Pt(100) and Pt(111) electrodes, respectively. A 0.1 M HClO_4_ solution was used as the blank solution.

To testify the generality of catalytic function for Pt oxides, electrooxidation of ethanol on Pt(110), Pt(100), and Pt(111) was observed as shown in Fig. 3. Figure 3a, b shows the comparison of voltammetric profiles generated for Pt(110) in 0.1 M perchloric acid (Fig. 3a) with different concentrations of ethanol (Fig. 3b), and the regions for PtOH_ads_, PtO_ads_, and α-PtO_2_ are marked in Fig. 3b. Peak M was observed above 0.60 V, which corresponded to the PtOH_ads_ potential region, and the current density was increased by adding ethanol into the solution. Peak N (~1.20 –1.40 V), which corresponded to α-PtO_2_, showed increased intensity upon the addition of ethanol. However, the valley bottom between peak M and peak N corresponded to the current peak for PtO_ads_ in a blank solution, indicating that PtO_ads_ does not act as the active catalytic center. The oxidation behavior of ethanol on Pt(100) and Pt(111), as shown in Fig. 3c–f, was consistent with the situation of Pt(110), which further verified the relationship between Pt oxides and the activity for the electrooxidation of ethanol.

### Potential-dependent product distribution

**Fig. 4:**
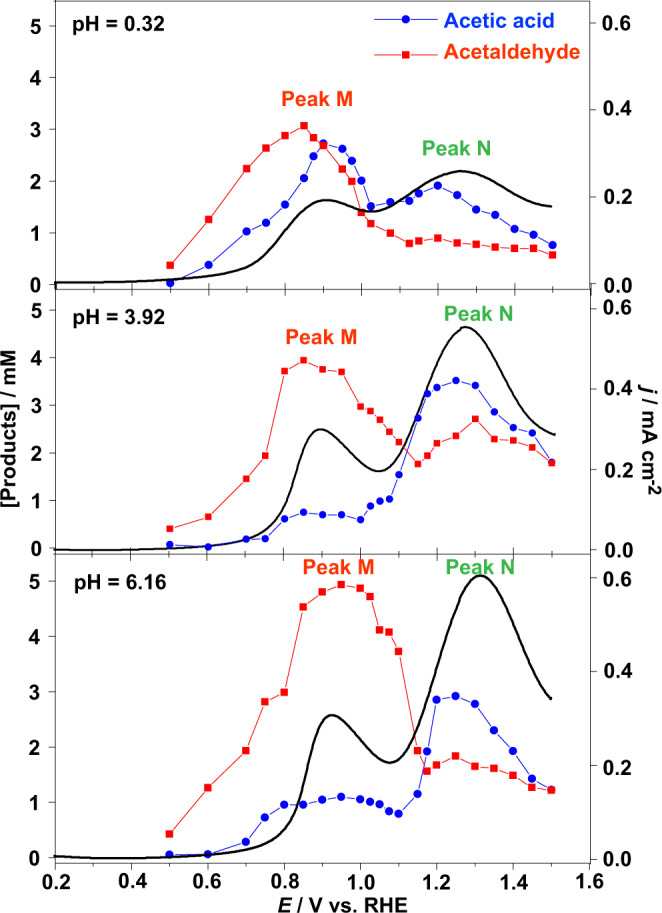
Association between curves of production concentrations and voltammogram at different pH during electrochemical oxidation of ethanol. pH = 0.32 (0.1 M ethanol at 0.5 M HClO_4_), pH = 3.92 (0.4999 M NaClO_4_ + 0.0001 M HClO_4_), and pH = 6.16 (0.5 M NaClO_4_). The red square (■) and blue dot (●) represent concentrations of acetaldehyde and acetic acid, respectively, determined by HPLC. The black curves stand for voltammetry profiles.

To further study the correspondence between Pt oxides and the activity of ethanol electrooxidation, chronoamperometry combined with HPLC was employed to monitor the voltammetry curves and product concentrations at different pH values, i.e., potential-dependent distribution of products for the electrooxidation of 0.1 M ethanol at different pH values are shown in Fig. 4. Both the concentrations of acetaldehyde and acetic acid approximately correspond to the current peaks, i.e., ethanol electrooxidation in peak M and peak N is derived from PtOH_ads_ and α-PtO_2_, respectively.

Notably, the height and area of current peak N were higher than those of peak M at all pH values, as shown in the voltammetry profiles of Fig. 4, indicating the surface PtO_2_ is a more powerful active center than PtOH_ads_. For every same pH, the concentration of acetaldehyde was higher than that of acetic acid at peak M; meanwhile, the concentration of acetic acid was higher than that of acetaldehyde at peak N, demonstrating that PtOH_ads_ and amorphous α-PtO_2_ particles are more likely to form acetaldehyde and acetic acid, respectively. With the increase of pH, current values in both peaks increased, the reason for which is that high pH favors the formation of Pt oxides. The acetaldehyde concentration at peak M was increased with the increase in pH, and conversely, the concentration of acetic acid decreased. However, non-monotonic pH dependence of the concentration of acetaldehyde and acetic acid at peak N was observed in an acidic medium, as shown in Fig. 4. In previous reports, PtO_2_ nanoparticles were shown to oxidize ethanol^46^ and favor complete oxidation to carbon dioxide^47^; this non-monotonic pH dependence possibly results from other products, such as carbon dioxide, from sufficient and more powerful PtO_2_ active centers in high pH.

### Atomic-level understanding and experimental explanation

**Fig. 5:**
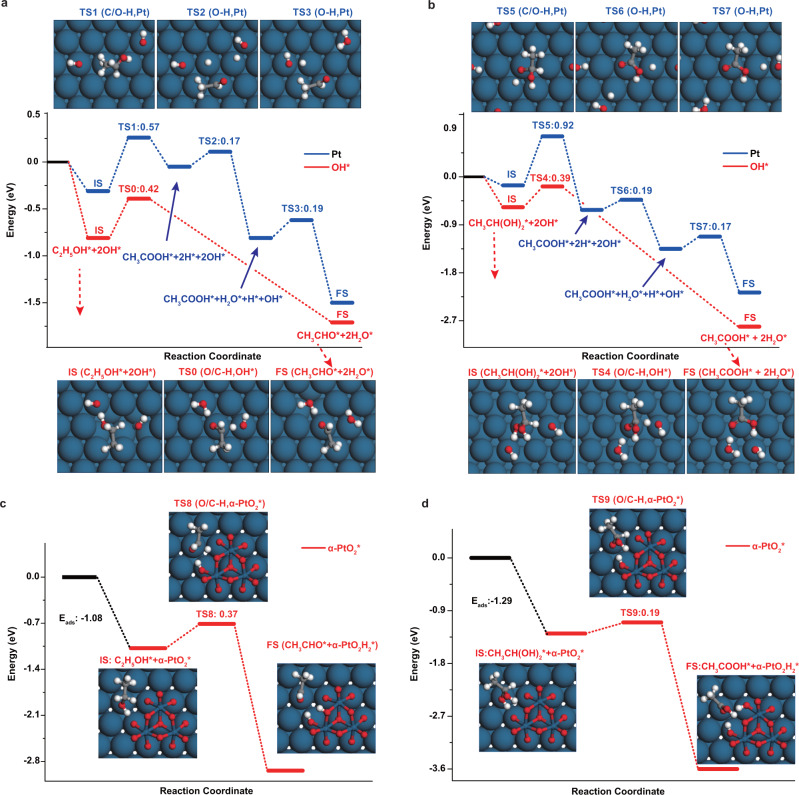
Energy profiles of the ethanol oxidation. **a**, **b** The comparisons of energy profiles for the formation of the acetaldehyde and acetic acid on Pt with (red path) and without (blue path) co-adsorbed OH. **c**, **d** Energy profiles for the formation of acetaldehyde and acetic acid on Pt with α-PtO_2_*, respectively. Surface structures of transition states (TS1-9) can be found in the above plots. Blue, gray, red, and white balls stand for Pt, C, O, and H atoms, respectively.

To explain the catalytic functions of the Pt oxides (PtOH_ads_ and PtO_2_), the energy profiles for ethanol electrooxidation on the Pt surface were calculated, as shown in Fig. 5 and Supplementary Tables 1–3 of the SI, in which the adsorption is represented by star (*). For PtOH_ads_, region A of the CV (Fig. 1a) corresponded to peak M for ethanol electrooxidation (Fig. 2), in which the hydroxyl groups were formed and adsorbed on the Pt surface (R1)^30^.

R1 $${{{{{\rm{Pt}}}}}}+{{{{{{\rm{H}}}}}}}_{2}{{{{{\rm{O}}}}}}\rightleftharpoons {{{{{{\rm{Pt}}}}}}-{{{{{\rm{OH}}}}}}}_{{{{{{\rm{ads}}}}}}}+{{{{{{\rm{H}}}}}}}^{+}+{{{{{{\rm{e}}}}}}}^{-}$$

It is well known that the interaction of ethanol with the surface of the Pt electrode is weak, and the presence of OH_ads_ would enable ethanol to enter the electrical double layer and adsorb on Pt. The adsorption energy (△E_ads_) of ethanol with co-adsorbed OH_ads_ is −0.81 eV, which is more advantageous than the energy without co-adsorbed OH (−0.30 eV), suggesting that OH_ads_ greatly enhanced ethanol adsorption on the Pt surface. Sheng et al.^48^ proposed that the OH_ads_ on a metal electrode, rather than the metal itself, provide the active centers and that concerted-like dehydrogenation caused by OH_ads_ acts as the dominant pathway for ethanol electrooxidation. This mechanistic pathway is used here for two reasons as follows. First, even that work^48^ calculated the role of water and adsorbed Hydroxyl in an alkaline medium, R1 for adsorbed hydroxyl universally accepted in acidic medium^20–22^, so this dehydrogenation mode can also be used to explain our experimental work about the active effect of PtOH_ads_ on ethanol electrooxidation in acidic solution (peak M in Fig. 2). Second, since PtOH_ads_ is distributed in large surface area (0.50 ML)^49^ and it can move in the surface through water exchange^33^, concerted-like dehydrogenation of substrate by PtOH_ads_ on surface to different products is used here as a favorable path for the full utilization of PtOH_ads_ and synergistic attraction of dihydrogen of ethanol with multiple PtOH_ads_^48^, although sequence dehydrogenation^33^ is also possible path. However, atomic-scale direct observation is needed for tracking surface dehydrogenation. For simple, feasible computation, two OH_ads_ sites were introduced into our model (marked as OH* in the energy profiles). A comparison of the energy profiles as well as the transition states for the formation of acetaldehyde on Pt with (red path) and without (blue path) co-adsorbed OH-induced dehydrogenation of ethanol is shown in Fig. 5a, and the corresponding thermodynamic data are shown in Supplementary Table 1. Ethanol undergoes dehydrogenation to form acetaldehyde, and the route depicted in blue lines represents ethanol dehydrogenation directly onto the Pt surface, which breaks the C-H bond and O-H bond (TS1, the activation barrier E_a_ = 0.57 eV) to form CH_3_CHO_ads_ and H_ads_. The H_ads_ combines with adjacent OH_ads_ generating H_2_O_ads_. The red route represents ethanol dehydrogenation using co-adsorbed OH_ads_, in which adsorbed ethanol undergoes concerted-like C-H bond breaking and O-H bond breaking to form acetaldehyde (TS0, E_a_ = 0.42 eV). The lower activation barrier suggests that OH_ads_ is beneficial for the dehydrogenation of ethanol (R2).

R2 $${{{{{\rm{Pt}}}}}}-{{{{{{\rm{CH}}}}}}}_{3}{{{{{{\rm{CH}}}}}}}_{2}{{{{{{\rm{OH}}}}}}}_{{{{{{\rm{ads}}}}}}}+{2{{{{{\rm{Pt}}}}}}-{{{{{\rm{OH}}}}}}}_{{{{{{\rm{ads}}}}}}}\rightleftharpoons {{{{{{\rm{Pt}}}}}}-{{{{{\rm{CH}}}}}}}_{3}{{{{{{\rm{CHO}}}}}}}_{{{{{{\rm{ads}}}}}}} +{2{{{{{\rm{H}}}}}}}_{2}{{{{{\rm{O}}}}}}+2{{{{{\rm{Pt}}}}}}$$

Acetaldehyde is quite active and easily hydrated to form the geminal diol (CH_3_CH(OH)_2_) in aqueous solution, with an equilibrium constant of 1.3, as shown in reaction (R3); this is accelerated dramatically by H^+^ cation in acidic medium or OH^−^ anion in alkaline medium, respectively^50^.

R3 $${{{{{{\rm{CH}}}}}}}_{3}{{{{{{\rm{CHO}}}}}}}_{({{{{{\rm{aq}}}}}})}+{{{{{{\rm{H}}}}}}}_{2}{{{{{{\rm{O}}}}}}}_{\left({{{{{\rm{aq}}}}}}\right)}\rightleftharpoons {{{{{{\rm{CH}}}}}}}_{3}{{{{{{\rm{CH}}}}}}({{{{{\rm{OH}}}}}})}_{2({{{{{\rm{aq}}}}}})}$$

A comparison of the energy profiles, as well as the transition states for the formation of acetic acid on Pt via the dehydrogenation of geminal diol with (red path) and without (blue path) facilitation of co-adsorbed OH_ads_, are shown in Fig. 5b, and the corresponding thermodynamic data are shown in Supplementary Table 2. The adsorption energy (△E_ads_) of geminal diol with co-adsorbed OH_ads_ is −0.57 eV, which is higher than the energy without co-adsorbed OH_ads_ (−0.16 eV), suggesting that OH_ads_ enhanced the adsorption of geminal diol on the Pt surface. According to previous work^43^, geminal diol can be dehydrogenated by PtOH_ads_ to form acetic acid, as shown in Fig. 5b (red path), for which the activation barrier was calculated to be 0.39 eV (TS4). Further, the activation barrier for the formation of acetic acid without co-adsorbed OH-induced dehydrogenation was calculated to be 0.92 eV (TS5). Therefore, the activation barrier of red path is lower than that of blue path, clarifying the catalytic function of PtOH_ads_ (R4).

R4 $${{{{{{\rm{Pt}}}}}}-{{{{{\rm{CH}}}}}}}_{3}{{{{{{\rm{CH}}}}}}({{{{{\rm{OH}}}}}})}_{2,{{{{{\rm{ads}}}}}}}+2\,{{{{{{\rm{Pt}}}}}}-{{{{{\rm{OH}}}}}}}_{{{{{{\rm{ads}}}}}}}\rightleftharpoons {{{{{{\rm{Pt}}}}}}-{{{{{\rm{CH}}}}}}}_{3}{{{{{{\rm{COOH}}}}}}}_{{{{{{\rm{ads}}}}}}} +2\,{{{{{{\rm{H}}}}}}}_{2}{{{{{\rm{O}}}}}}+2{{{{{\rm{Pt}}}}}}$$

The reaction rate of R3 for producing geminal diol depends on pH^50^. According to (R2-R4), when pH increases in acidic solution, slower reaction R3 causes higher selectivity of acetaldehyde over acetic acid, which agrees with experimental results of peak M in Fig. 4, i.e., the favorable dehydrogenation path of geminal diol (R4) in low pH medium can be experimentally supported. According to the above calculation results, mechanisms for ethanol oxidation at PtOH_ads_ active centers were defined (R1-R4). On the one hand, OH_ads_ strengthens the adsorption of ethanol on the Pt surface. On the other hand, the presence of OH_ads_ reduces the activation barrier of ethanol electrooxidation, thereby facilitating the dehydrogenation of ethanol. The comparison of the two paths can better explain the essential role of PtOH_ads_ as active center^28,29^.

When the applied potential in Region B increased to the range from 0.95 to 1.05 V, PtOH_ads_ was further oxidized to PtO_ads_, as shown in Fig. 1a, through reaction (R5)^51^. PtO_ads_ showed no peak of current and product for ethanol electrooxidation, as shown in Figs. 2–4, due to the structural stability from the shorter bond between O_ads_ and Pt^22^ and PE-induced subsurface oxygen (O_*sub*_)^23^, causing this oxide to be inactive.

R5 $${{{{{{\rm{Pt}}}}}}-{{{{{\rm{OH}}}}}}}_{{{{{{\rm{ads}}}}}}}\rightleftharpoons {{{{{{\rm{Pt}}}}}}-{{{{{\rm{O}}}}}}}_{{{{{{\rm{ads}}}}}}}+{{{{{{\rm{H}}}}}}}^{+}+{{{{{{\rm{e}}}}}}}^{-}$$

α-PtO_2_ formed in region C (Fig. 1a and Fig. 3) above 1.15 V (R6-R7)^26,42^, which resulted in a corresponding peak for ethanol electrocatalytic oxidation, as indicated by Peak N in Figs. 2–4.

R6 $$2\,{{{{{{\rm{Pt}}}}}}-{{{{{\rm{OH}}}}}}}_{{{{{{\rm{ads}}}}}}}\rightleftharpoons {{{{{{\rm{\alpha }}}}}}-{{{{{\rm{PtO}}}}}}}_{2}+2\,{{{{{{\rm{H}}}}}}}^{+}+2\,{{{{{{\rm{e}}}}}}}^{-}+{{{{{\rm{Pt}}}}}}$$

R7 $${{{{{{\rm{Pt}}}}}}-{{{{{\rm{O}}}}}}}_{{{{{{\rm{ads}}}}}}}+{{{{{{\rm{H}}}}}}}_{2}{{{{{\rm{O}}}}}}\rightleftharpoons {{{{{{\rm{\alpha }}}}}}-{{{{{\rm{PtO}}}}}}}_{2}+2\,{{{{{{\rm{H}}}}}}}^{+}+2\,{{{{{{\rm{e}}}}}}}^{-}$$

Here, the active role of α-PtO_2_ was explored with the catalysis model shown in Supplementary Fig. 4; the locally covered α-PtO_2_ micro-particle was used for ethanol electrooxidation^52,53^, since approximately 50% of the Pt atoms formed disordered oxides, 20% of the surface atoms were located on place-exchange sites, and 30% remained in regular surface sites during the Pt oxidation process^54^.

The energy profiles for ethanol oxidation in the presence of α-PtO_2_ are shown in Fig. 5c, d, and the calculation results are shown in Supplementary Table 3. The IS (initial state) of ethanol oxidation in the presence of α-PtO_2_ with an adsorption energy of −1.08 eV (−0.81 eV with OH_ads_) indicates that α-PtO_2_ more greatly strengthens the adsorption of ethanol on the Pt surface than PtOH_ads_. The black path in Fig. 5c shows the process of concerted-like ethanol dehydrogenation (the O-H and C-H band cleavage) by α-PtO_2_ to form acetaldehyde, of which the activation barrier (TS8, red line) is 0.37 eV. In Fig. 5d, the adsorption energy of geminal diol in the presence of α-PtO_2_ is −1.29 eV, much higher than that in the presence of PtOH_ads_ (−0.57 eV), suggesting that α-PtO_2_ results stronger adsorption of geminal diol on the Pt surface than PtOH_ads_. Then geminal diol undergoes concerted-like dehydrogenation of C-H and O-H bonds with an energy barrier of 0.19 eV (TS9) to form CH_3_COOH_ads_. The activation barrier of ethanol dehydrogenation to acetaldehyde and acetic acid by α-PtO_2_ is lower (0.37 and 0.19 eV) than that by OH_ads_ (0.42 and 0.39 eV), indicating that the α-PtO_2_ is a more active center than PtOH_ads_ to benefit the oxidation of ethanol to high concentration of acetic acid, which can explain that high selectivity of acetic acid at peak N in high potential range (Fig. 4). When pH increase, more PtO_2_ particles on Pt surface would form, and excess PtO_2_ could oxidize ethanol to other carbon products of high oxidation state such as carbon dioxide^42^, resulting in non-monotonic pH dependence of the concentration of acetaldehyde and acetic acid at peak N as shown in Fig. 4.

In conclusion, the relationship between Pt oxides and ethanol electrooxidation activity in perchlorate acid was obtained, i.e., surface PtOH_ads_ and α-PtO_2_, as active centers, catalyze ethanol oxidation; in contrast, surface PtO_ads_ is inactive and does not generate corresponding peaks for current density and product concentration. A Pt oxides-mediated reaction mechanism for ethanol oxidation on Pt catalyst was developed with the help of DFT computation, in which Pt oxide-mediated dehydrogenation and hydrated reaction intermediate, i.e., geminal diol, can perfectly explain the experimental results such as pH dependence of product selectivity and more active α-PtO_2_ than PtOH_ads_. This work distinguished the functions of different Pt oxides in ethanol electrochemical oxidation and was not limited to consideration of surface PtOH_ads_ as in past studies (the maximum applied potential in most previous work did not exceed 1.10 V)^28–33^. These results can be extended to other electrodes, such as metal oxides^16^, alloys^55^, MOFs^56^, and perovskites^57^, and oxidations of other substrates.

## Methods

### Electrochemical measurements

All solutions were prepared with ultrapure water (Milli-Q system, 18.2 MΩ•cm). The chemicals used for solution preparation were perchloric acid (Suprapur, Merck) and sodium perchlorate (Suprapur, Merck). All other chemicals were of the highest commercially available purity (ethanol, acetaldehyde, and acetic acid) and were used without further purification. All solutions in the electrochemical experiments were deoxygenated with N_2_ (99.999%). Before each measurement, all equipment in contact with the electrolyte and electrodes was rinsed with distilled water, treated with diluted KMnO_4_ solution for at least 12 h, and diluted H_2_O_2_ solution for 8 h. Finally, they were thoroughly rinsed with deionized water.

The working electrode was prepared by depositing 4 µL of an aqueous solution containing 2 mg mL^−1^ polyoriented Pt nanoparticles (Premetek) onto a glassy carbon electrode (2.0 mm in diameter) embedded in a Teflon insulator and 2 µL of a 5 wt.% Nafion (Sigma–Aldrich) dispersion in ethanol was added to guarantee powder adhesion. The parameters, TEM images, and the X-ray diffraction pattern (XRD) of commercial Pt nanoparticles are shown in Supplementary Table 4, Supplementary Figs. 5 and 6. The single-crystal electrodes were annealed in an N-butane flame and cooled in a hydrogen and argon atmosphere. Before the experiments, cyclic voltammograms from 0.05 to 1.50 V with a scan rate of 50 mV s^−1^ in a nitrogen-purged supporting electrolyte were performed to ensure the cleanliness of the systems. The electrochemically active area of Pt was measured using the charge involved in the so-called hydrogen adsorption/desorption region (between 0.05 and 0.4 V) from the cyclic voltammograms. In order to quantify the proportion of Pt(100), Pt(111), and Pt (110) domains on the Pt nanoparticles, irreversible adsorption of Germanium (IV) and Bismuth (III) was performed by spontaneous deposition from a 10^−2^ M solution of GeO_2_ (Aladdin 99.999%) in 1.0 M NaOH and a saturated solution of Bi_2_O_3_ (Aladdin 99.999%) in 0.5 M H_2_SO_4_, respectivly^58^. After deposition, the electrode surface was rinsed with water and immersed in the electrochemical cell at 0.1 V with the droplet attached for voltammetry scanning. In Supplementary Fig. 2a, Bismuth (III) adsorbed on (111) sites shows a redox peak (R8) at around 0.63 V, whose charge is proportional to the number of (111) sites, as shown in Eq. (E1)^59^:

R8 $${{{{{{\rm{Pt}}}}}}}_{3}{{{{{{\rm{Bi}}}}}}}_{{{{{{\rm{ads}}}}}}}+{2{{{{{\rm{H}}}}}}}_{2}{{{{{\rm{O}}}}}}={{{{{{\rm{Pt}}}}}}}_{3}{{{{{{\rm{Bi}}}}}}({{{{{\rm{OH}}}}}})}_{2,{{{{{\rm{ads}}}}}}}+{2{{{{{\rm{H}}}}}}}^{+}+{2{{{{{\rm{e}}}}}}}^{-}$$

E1 $${{{{{{\rm{q}}}}}}}_{{{{{{\rm{Bi}}}}}}}=0.64\pm {0.03{{{{{\rm{q}}}}}}}_{{{{{{\rm{Pt}}}}}}(111)}$$

In which q represents charge density, the area (A) of Pt(111) can be obtained by (E2):

E2 $${{{{{{\rm{A}}}}}}}_{{{{{{\rm{Pt}}}}}}(111)}={{{{{{\rm{q}}}}}}}_{{{{{{\rm{Pt}}}}}}(111)}/220$$

In Supplementary Fig. 2b, Germanium (IV) adsorbed on (100) sites shows a redox peak (R9) at around 0.48 V, whose charge is proportional to the number of (100) sites, as shown in Eq. (E3)^60^:

R9 $${{{{{{\rm{Pt}}}}}}}_{4}{{{{{{\rm{Ge}}}}}}}_{{{{{{\rm{ads}}}}}}}+{{{{{{\rm{H}}}}}}}_{2}{{{{{\rm{O}}}}}}={{{{{{\rm{Pt}}}}}}}_{4}{{{{{\rm{GeO}}}}}}+{2{{{{{\rm{H}}}}}}}^{+}+{2{{{{{\rm{e}}}}}}}^{-}$$

E3 $${{{{{{\rm{q}}}}}}}_{{{{{{\rm{Ge}}}}}}}=0.56\pm {0.03{{{{{\rm{q}}}}}}}_{{{{{{\rm{Pt}}}}}}(100)}$$

The area (A) of Pt(100) can be obtained by (E4):

E4 $${{{{{{\rm{A}}}}}}}_{{{{{{\rm{Pt}}}}}}(100)}={{{{{{\rm{q}}}}}}}_{{{{{{\rm{Pt}}}}}}(100)}/209$$

The proportion of Pt(100) and Pt(111) domains was obtained by the ratio of (100) and (111) area to the total electrode area, respectively; the rest proportion is Pt(110) domain^61^.

All experiments were conducted at 25 °C using a computer-controlled electrochemical workstation (Bio-Logic VSP-300) in a conventional three-electrode glass cell deaerated by nitrogen (99.999%), including a 4.0 cm diameter platinum ring counter electrode (CE) placed 1.5 cm below the WE and a J-shaped reversible hydrogen electrode (RHE) filled with 0.50 M HClO_4_ or H_2_SO_4_ solution that served as the reference electrode. The RHE was placed in a fixed position relative to the WE surface, and the electrolyte solution volume was 50 mL.

### In situ SERS experiments

Raman spectra were recorded with an HR-800 (Jobin Yvon-Horiba, France) spectrometer integrated with a confocal microscope. A schematic of the in situ surface-enhanced Raman spectroscopy is shown in Supplementary Fig. 7a. The spectra were obtained by excitation with an air-cooled frequency-doubled ND YAG laser emitting a wavelength of 532 nm. The working electrode was prepared by depositing 4 µL of a 2 mg mL^−1^ platinum nanoparticle water suspension onto a polycrystalline platinum disk electrode (2.0 mm in diameter) embedded in a Teflon insulator. The suspension was deposited with four subsequent aliquots of 1 µL. The WE was then mounted on a 20-mL electrochemical polytetrafluoroethylene (PTFE) cell designed to acquire in situ Raman spectra. A fused silica window separated the microscope objective from the 0.5 M HClO_4_ electrolytic solution. All potentials referred to the RHE electrode and the potential was held constant for approximately 15 min during each Raman measurement.

### Chronoamperometry combined with HPLC

These experiments were performed in an electrochemical cell with 80 mL of 0.5 M solution containing HClO_4_ and NaClO_4_ and 0.1 M ethanol deaerated with nitrogen. The working electrode was prepared by depositing 30 µL of 2 mg mL^−1^ Pt nanoparticle suspension onto a glassy carbon electrode (6.0 mm in diameter) embedded in a Teflon insulator. The suspension was deposited into six subsequent aliquots of 5 µL, and 10 µL of 5 wt.% Nafion dispersion was added. Before sampling, the WE was immersed in 0.5 M HClO_4_ and NaClO_4_ solution with 0.1 M ethanol under potentiostatic control at different potentials for 20 min to ensure sufficient reaction. Then, the applied potential was increased to a specific value for the next sampling. The products on the interface were almost completely removed during sampling. Hence, pH was changed slightly even weak-buffering HClO_4_–NaClO_4_ or H_2_SO_4_–Na_2_SO_4_ solutions were used, e.g., the pH of a solution changed from 6.16 to 6.02 after one day of reaction, i.e., sampling shows the function of pH-buffering. To compare the voltammograms with current densities (j) for different applied potentials (E) with chronoamperometry, the j-t and j-E plots at the sampling potentials are shown in Supplementary Fig. 8. The trend for the j-E curve in Supplementary Fig. 8b was consistent with the CV curves for ethanol electrooxidation (Fig. 2), indicating that it is feasible to sample by chronoamperometry and determine the relationships between the CV curve and product concentrations. A micrometer-sized sampling tip (diameter, 0.3 mm) was placed close (10 μm) to the surface of the WE for rapid sampling (with an auxiliary pump, Dionex™). In the case of ethanol electrooxidation, the optimal flow rate was found to be 0.1 mL min^−1^. After collection, all samples were analyzed by HPLC (Agilent 1260) with a Phenomenex FMH-1138-KONU column (300 × 7.8 mm, 10 μm) and a refractive index detector (RID). The samples were acquired by injecting 5 μL of the solution into the HPLC system. The mobile phase of the ethanol system was 5 mM H_2_SO_4_. The temperature of the column was maintained at 60 °C in a column oven, and the separated compounds were detected at 35 °C. Detailed implementation for the combination of fraction collection from the electrode surface and subsequent sample analysis by HPLC is provided elsewhere^62^. A schematic of the online sample collection with the AXP pump combined with the HPLC system is shown in Supplementary Fig. 7b.

### Computational methods

The calculations with spin-polarized DFT were performed with the Vienna Ab-Initio Simulation Package (VASP)^63^. The Kohn-Sham wave functions were expanded in a plane-wave basis set. The cutoff energy was set to 400 eV. The projector-augmented wave (PAW) method and PBE potential for the exchange-correlation function were used^64^. A (3 × 3 × 1) Monkhorst-Pack k-point sampling method was used^65^. All atoms were allowed to relax until the forces and energies fell below 0.02 eV Å^−1^ and 10^−5 ^eV, respectively. We employed a Pt(111) surface with a p(4×4) periodic slab of four atomic layers including 64 atoms for OH_ads_ on Pt surface, and another Pt(111) surface with a p(4 × 4) periodic slab of two atomic layers including 32 atoms for α-PtO_2_ on Pt surface referring to previous work^52,53^, where the bottom two layers of atoms were fixed in the slab while the atoms of the rest were relaxed during all optimization processes. A vacuum region of 15 Å was created to ensure negligible interactions between mirror images. Climbing image nudged elastic band (CINEB) and Dimer were used for studying the transition state. The convergence of forces was set to 0.03 eV Å^−1^ using a modified VASP. The structures of ethanol, acetaldehyde, acetic acid, water, and other molecules were optimized in a 15 Å vacuum square box.

## Supplementary information

Supplemental Material

## Data Availability

The data that support the findings of this study are available from the corresponding author upon reasonable request.
