# Supplementary material for: Relationship between oxide identity and electrocatalytic activity of platinum for ethanol electrooxidation in perchlorate acidic solution
Source: Commun Chem. 2023 May 29;6:101. doi: 10.1038/s42004-023-00908-3 (PMC10227044; doi:10.1038/s42004-023-00908-3)
Supplement: Supplementary file 1 — Supplemental Material [file 42004_2023_908_MOESM1_ESM.pdf]

Supplementary Information

Relationship between oxide identity and electrocatalytic activity of Platinum for ethanol electrooxidation in perchlorate acidic solution

Xinyu You,[1,5] Jiaxing Han,[1,5] Vinicius Del Colle,[2] Yuqiang Xu,[1] Yannan Chang,[1] Xiao Sun,[1] Guichang Wang,[3] Chen Ji,[3] Changwei Pan,[1*] Jiujun Zhang,[1,4*] and Qingyu Gao[1*]

[1] College of Chemical Engineering, China University of Mining and Technology, Xuzhou 221116, People's Republic of China

[2] Department of Chemistry, Federal University of Alagoas-Campus Arapiraca, Av. Manoel Severino Barbosa s/n, Arapiraca, AL, 57309-005, Brazil

3 Department of Chemistry, Nankai University 300071, Tianjin, People's Republic of China

[4] School of materials science and engineering, Fuzhou University, Fuzhou 350108 , People's Republic of China

[5]These authors contributed equally: Xinyu You, Jiaxing Han.

[*]Email: gaoqy@cumt.edu.cn, jiujun.zhang@i.shu.edu.cn, and cwpan2002@126.com

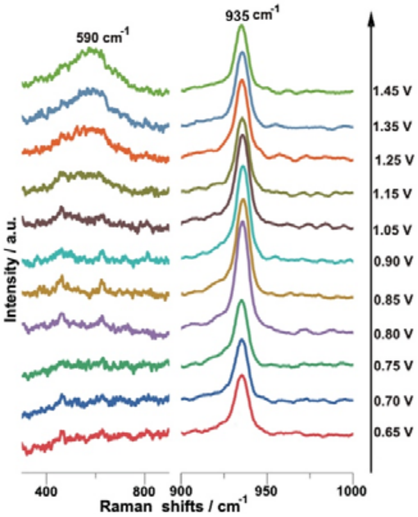

Supplementary Figure 1 | In situ SERS at the indicated potentials in 0.1 M perchloric acid solution.

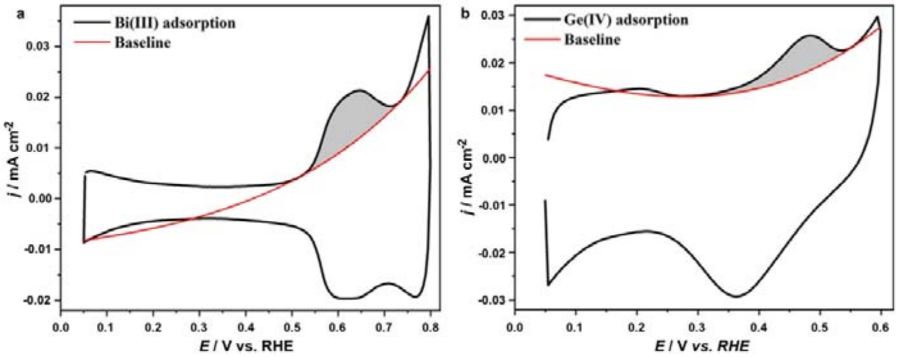

Supplementary Figure 2 | Quantification of Pt(111) and Pt(100) domains. a, Bi(III) adsorption curve for quantification of Pt(111) domains. **b**, Ge(IV) adsorption curve for quantification of Pt(100) domains. The red is the baseline.

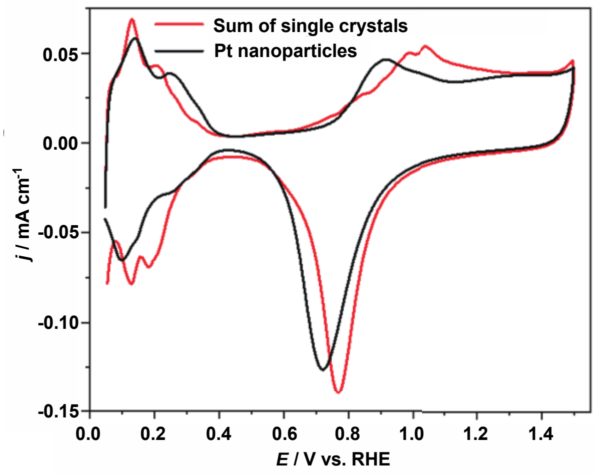

Supplementary Figure 3 | Experimental and calculated CV profiles in 0.10 M $HClO_4$ solution. a, Experimental CV profile on Pt nanoparticles. b, Sum of CV profiles according to individual content of three basal crystal planes, i.e. 11.69 % Pt(100), 16.52 % Pt(111), and 71.79 % Pt(110). The red line and black line denote the relative ratio sum of single-crystal current and CV on Pt nanoparticles, respectively.

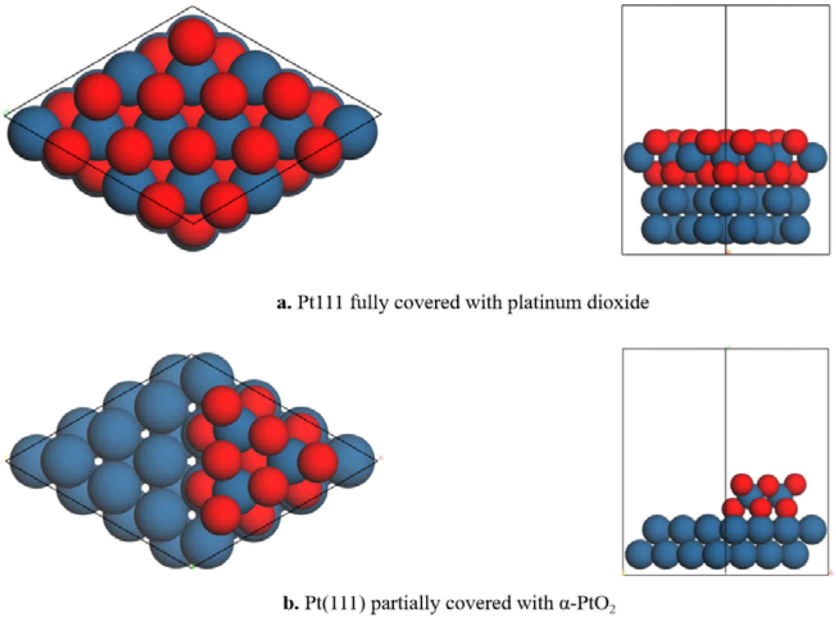

Supplementary Figure 4 | Completely covered (a) and locally covered (b) model structure of α-PtO_2.

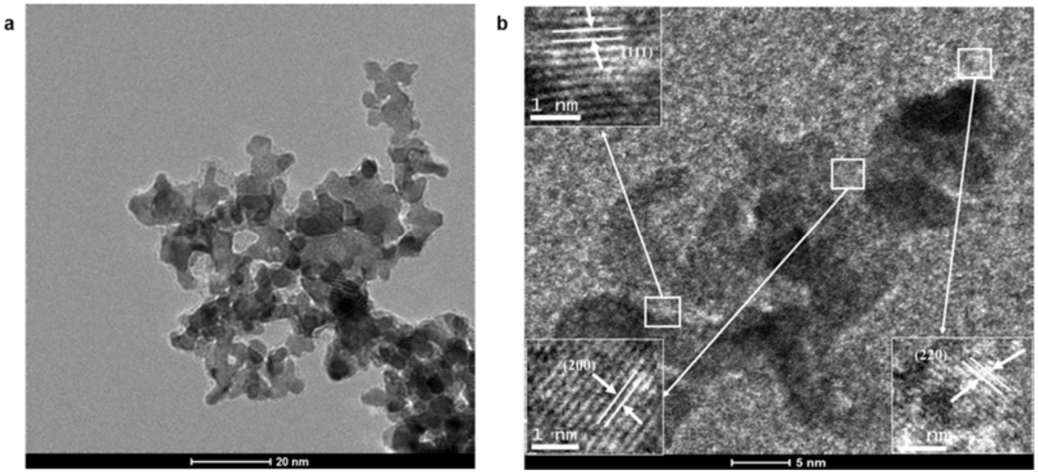

Supplementary Figure 5 | TEM images of Pt nanoparticles. **a**, Regular and **b**, high-resolution TEM images of the commercial Pt nanoparticles used in this work. The (200) and (220) crystal faces are parallel to the (100) and (110) faces respectively and have the same arrangement of atoms in the faces.

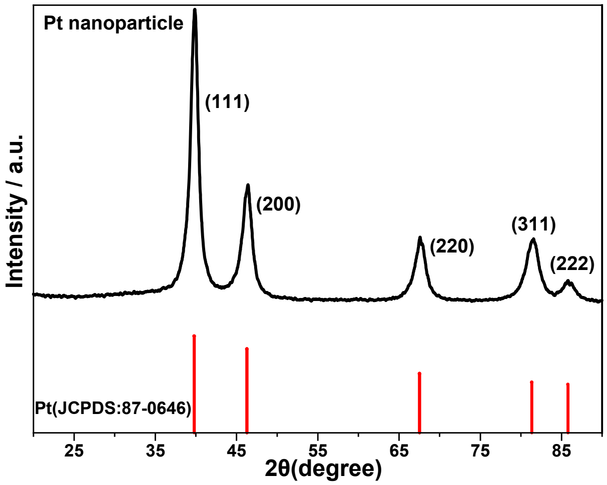

Supplementary Figure 6 | XRD pattern of Pt nanoparticles. (200), (220) and (222) are the second-order diffraction of (100), (110) and (111), respectively; (311) is composed of 2 (111) terraces and (100) step.

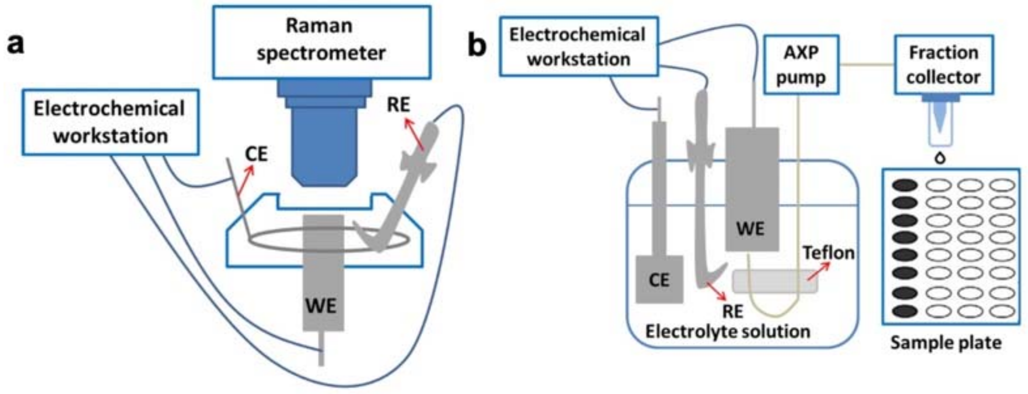

Supplementary Figure 7 | Schematic of in situ SERS and EC-HPLC. a, Schematic of the in situ surface-enhanced Raman spectroscopy. **b**, Schematic of the on-line sample collection with AXP pump conjunct with HPLC system. WE: working electrode, RE: reference electrode, CE: counter electrode.

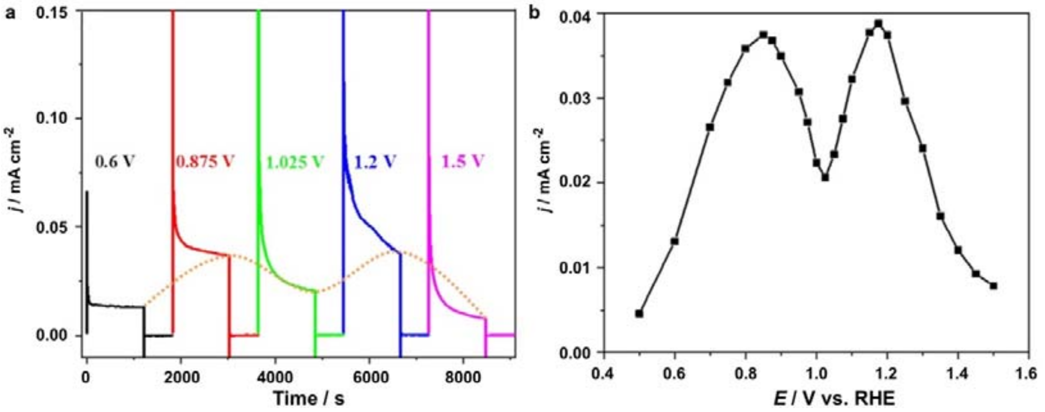

Supplementary Figure 8 | Chronoamperometry and its quasi-steady j-E curve. a, The *j*-t plots of selected potentials by chronoamperometry. **b**, The trend of current density at sampling potentials in the experimental measurements of chronoamperometry. The solution is 0.5 M $HClO_4$ with 0.1 M ethanol.

Supplementary Table 1. Comparison of activation barriers (Ea) and reaction energy (ΔE) in formation of acetaldehyde with and without co-adsorbed OH* (Fig. 5a)

Reaction	Catalytic center	Ea/eV	ΔE/eV
CH_3CH_2OH*+* → CH_3CHO* + 2H*	Pt	0.57	0.26
CH_3CH_2OH* + 2OH* → CH_3CHO* + H_2O* + H_2O+*	OH*	0.42	-0.90

Supplementary Table 2. Comparison of activation barriers (Ea) and reaction energy (ΔE) in formation of acetic acid with and without OH* (Fig. 5b)

Reaction	Catalytic center	Ea/eV	ΔE/eV
$CH_3CH(OH)_2$* +*→CH_3COOH* + 2H*	Pt	0.92	-0.46
$CH_3CH(OH)_2$* + 2OH* → CH_3COOH* + H_2O* +H_2O+*	OH*	0.39	-2.24

Supplementary Table 3. Comparison of activation barriers (Ea) and reaction energy (ΔE) in ethanol dehydrogenation with and without α-PtO_2 (Fig. 5c, d)

Reaction	catalytic center	Ea/eV	Δ E/eV
CH_3CH_2OH* + PtO_2 → CH_3CHO* + $Pt(OH)_2$*	PtO_2	0.37	-1.86
CH_3CH_2OH* + 2OH*→ CH_3CHO*+ $Pt(OH)_2$	Pt-O_2*	0.19	-2.31

Supplementary Table 4. Performance parameters of nanoparticles

**The parameters of Pt nanoparticles**	
Platinum content	＞ 98 %
Specific surface area	45 ~ 52 m^2/g
Bulk density	0.6 ~ 0.9 g/mL
Mean diameter	5.0 ~ 7.0 nm
Total metallic impurities	＜ 500 ppm
